# Relapse of Eosinophilic Esophagitis on Dupilumab

**DOI:** 10.1097/PG9.0000000000000273

**Published:** 2022-11-08

**Authors:** Matthew A. Buendia, Yash A. Choksi, Girish Hiremath

**Affiliations:** From the * Division of Pediatric Gastroenterology, Hepatology and Nutrition, Department of Pediatrics, Vanderbilt University Medical Center, Nashville, TN; † Division of Gastroenterology, Hepatology and Nutrition, Department of Medicine, Vanderbilt University Medical Center, Nashville, TN; ‡ Tennessee Valley Health System, Veterans Affairs, Nashville, TN.

**Keywords:** eosinophilic esophagitis, dupilumab, topical steroids

## Abstract

Dupilumab is approved for the treatment of eosinophilic esophagitis (EoE). We report a teenager with difficult-to-treat EoE on topical corticosteroids (TS) who achieved clinical and histological remission when initiated on dupilumab for a primary indication of atopic dermatitis. However, when his TS were weaned after achieving remission, his disease relapsed with worsening of his dysphagia and a peak eosinophilic count (PEC) of 55 eosinophils per high power field (eos/hpf). Upon restarting TS to his ongoing dupilumab, symptoms fully resolved, and he achieved histologic remission (PEC 10 eos/hpf). This report underscores the: (1) importance of longitudinal monitoring for EoE patients on dupilumab, (2) unmet need for guidance on how to transition EoE patients on traditional therapies to dupilumab, and (3) need for longitudinal follow-up data on dupilumab to help personalize therapy for EoE patients.

## INTRODUCTION

Eosinophilic esophagitis (EoE) is a complex T helper-2 (Th-2)-driven inflammatory disorder. An allergen mediated condition, it is characterized by symptoms of esophageal dysfunction and an intense eosinophilic inflammation (defined as a peak eosinophil count [PEC] of ≥15 eosinophils per high power field [eos/hpf]).^1^ Persistent and sub-optimally controlled eosinophilic inflammation can lead to dysphagia, chest pain, and recurrent esophageal food impactions requiring endoscopic intervention.^2^ Medications such as proton pump inhibitors (PPI) and topical corticosteroids (TS), and elimination of dietary allergens are conventionally used to induce remission (defined as <15 eos/hpf).^3^ However, while PPI and TS can induce clinical improvement and histologic remission in about half of EoE patients, the disease can recur after their withdrawal,^4^ and a dietary elimination therapy can be burdensome, often has very poor compliance, and can negatively impact the patient’s quality of life.^5^

Recently, dupilumab, a humanized monoclonal antibody that inhibits the interleukin (IL)-4 receptor alpha chain augmenting IL-4 and IL-13 signaling, major mediators of Th-2 inflammation, became the first United States Food and Drug Administration–approved treatment for EoE in children ≥12 years of age.^6^ Data from phase 2 and phase 3 multicenter clinical trials show that dupilumab when compared to placebo significantly improved clinical symptoms, endoscopic signs, esophageal eosinophil counts, and histologic alterations in patients with EoE who failed PPI therapy.^7,8^ In these studies, patients with EoE who were refractory to TS therapy were not included. Additionally, reports illustrate the utility of dupilumab to manage difficult-to-treat EoE patients.^9^ We present an adolescent with EoE who had previously failed conventional therapies, achieved remission on dupilumab but then relapsed again while maintained on it.

## CASE REPORT

A 17-year-old Caucasian male was transferred to our multidisciplinary eosinophilic gastrointestinal diseases clinic from an outside hospital for management of his EoE. He had been diagnosed with EoE approximately 10 years earlier and had already failed skin prick test directed and empiric dietary elimination. Dysphagia and chest pain were his primary symptoms, and he had 2 prior episodes of esophageal food impactions requiring endoscopic removal of food bolus. He was on a PPI and swallowed fluticasone at presentation to our clinic. He did not have a history of esophageal dilation. He also had concomitant atopic dermatitis, asthma, and allergic rhinitis managed by an allergist with cetirizine, nasal fluticasone, and albuterol as needed. His chest pain evaluation was negative for cardiopulmonary causes and was attributed to his longstanding EoE.

**FIGURE 1.**
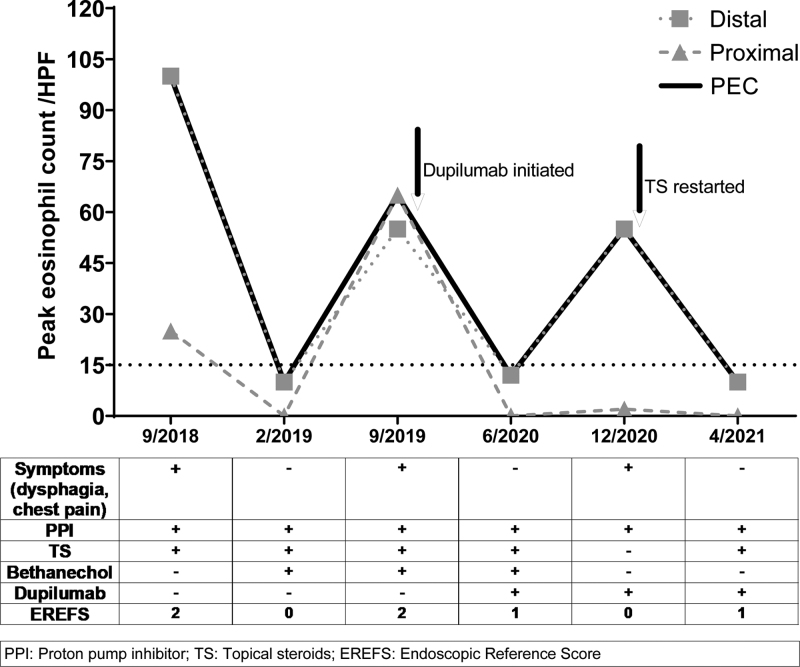
Peak eosinophil count per high powered field over time (arrows indicate initiation of therapy). EREFS = EoE Endoscopic Reference Score; HPF = high power field; PEC = peak eosinophilic count; PPI = proton pump inhibitors; TS = topical corticosteroids.

At our center, the swallowed fluticasone was discontinued, and he was initiated on budesonide slurry (0.5 mg/2 mL respule; 2 respules mixed in 2 teaspoons of applesauce, 2 times per day).^1^ He was continued on PPI and ongoing anti-allergy medications. Even while reporting high compliance to his medications, he was unable to achieve sustained improvement of esophageal symptoms and of histologic remission of his EoE. His PEC ranged between 65 and 123 eos/hpf over 15 months at our center, indicating a difficult to control intense eosinophilic inflammation (Fig. 1).

High-resolution esophageal manometry revealed frequent failed peristalsis per the Chicago Classification, so bethanechol was added to his regimen. However, this did not improve his esophageal symptoms. Separately, he was having worsening of his dermatitis. Following discussions with our eosinophilic gastrointestinal disease clinic, his allergist started him on dupilumab (600 mg subcutaneously [S.C.] loading dose followed by 300 mg S.C., every 2 weeks) to manage his severe dermatitis. This resulted in notable improvement in his dermatitis and asthma. Interestingly, he also experienced complete resolution of his esophageal symptoms and achieved histologic remission (PEC: 12 eos/hpf) after 4 months.

As his EoE was in remission, he was weaned off budesonide slurry and bethanechol but continued on dupilumab and PPI with a plan to re-assess EoE activity in 6 months.

**FIGURE 2.**
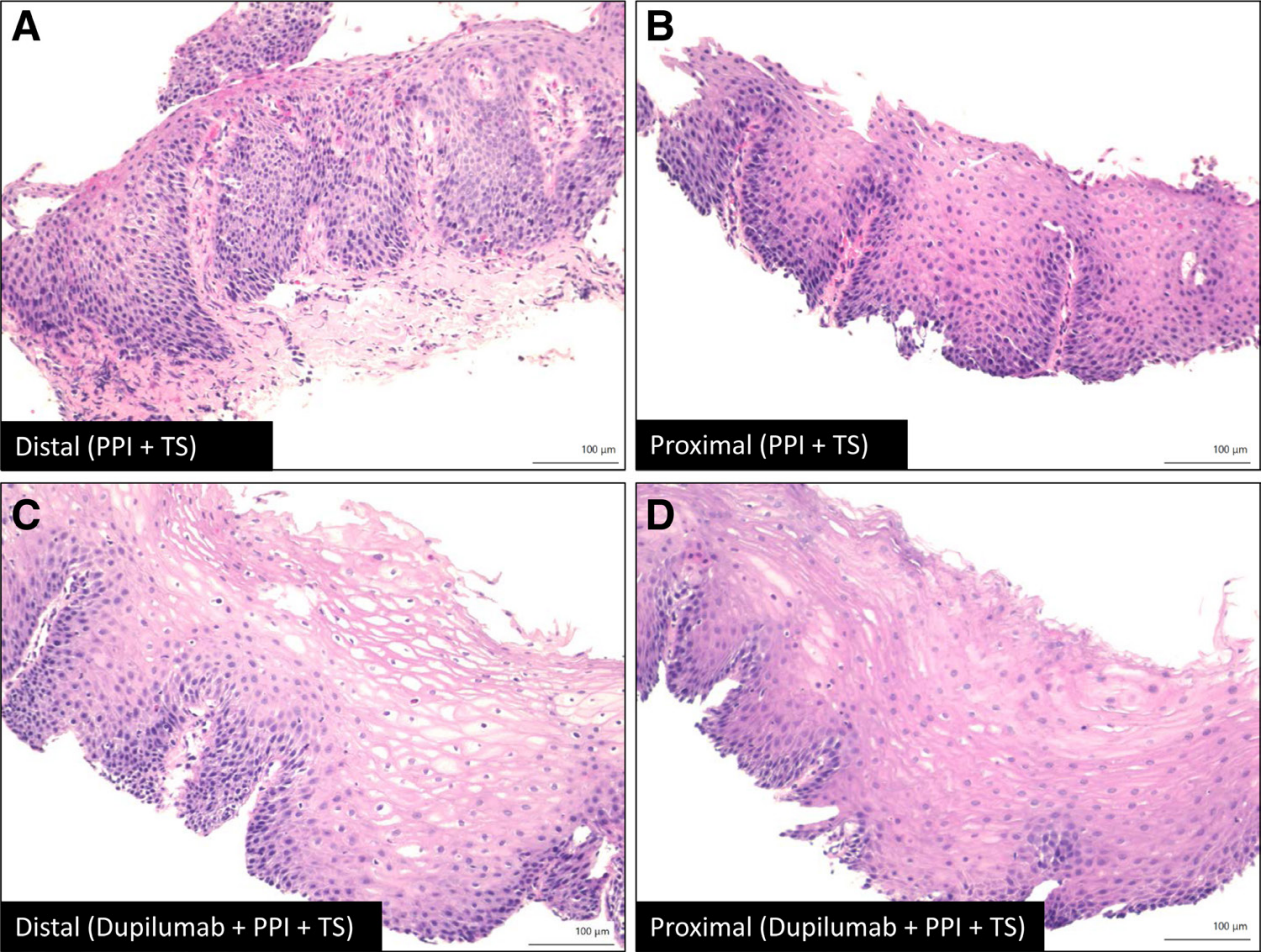
Representative images of the esophageal biopsies (H&E stain): (A) and (B) Distal and proximal sites, respectively, on PPI and TS showing basal cell hyperplasia and intense eosinophilia; (C) and (D) Distal and proximal, respectively, on dupilumab, PPI and TS illustrating significant improvement in basal cell hyperplasia and eosinophilia. PPI = proton pump inhibitors; TS = topical corticosteroids.

Approximately 3–4 weeks before his scheduled esophagogastroduodenoscopy (EGD), he began experiencing dysphagia. Esophageal biopsies revealed a PEC of 55 eos/hpf, indicating that his EoE had relapsed while he was on dupilumab and PPI. Subsequently, low dose budesonide slurry (0.5 mg/2 mL mixed with 1 teaspoon of applesauce once a day) was added. Following this, at his 4-month follow-up, he reported complete resolution of his esophageal symptoms, and he once again achieved histologic remission (PEC: 10 eos/hpf) (Fig. 2). At this point, his EoE care was transitioned to an adult gastroenterologist.

## DISCUSSION

We present a difficult-to-treat adolescent EoE patient who initially achieved clinical and histologic remission on dupilumab when paired with conventional therapies (both PPI and TS). However, his disease rapidly relapsed when weaning TS though he was still on dupilumab and PPI. He then reattained clinical and histological remission when TS was added back. This case raises several important points related to application of dupilumab in the future. First, it underscores the importance of longitudinal monitoring of EoE on dupilumab, both clinically and histologically, in routine practice. Second, additional research is needed on how to transition EoE patients on conventional therapies to dupilumab, with particular attention to the concurrent use of conventional therapies. Third, our patient received dupilumab per the atopic dermatitis protocol. For EoE, the United States Food and Drug Administration has approved dupilumab 300 mg every week, S.C. Thus, investigation of the difference in dose and frequency of dupilumab and how this plays a role in long-term control of EoE is needed. Thus, our experience identifies knowledge gaps directly relevant to the use of dupilumab in routine clinical practice. For instance, what is the non-response and/or relapse rate of EoE in patients on dupilumab monotherapy and combination therapy and is there a way to predict responders (or non-responders) to dupilumab to personalize their care. Even though there are limited data on esophageal dysmotility in pediatric EoE patients, another question pertinent to our case is: can failed peristalsis (or esophageal dysmotility) on high-resolution esophageal manometry serve as a predictor of response to dupilumab? These points may also be relevant to other biologics that are currently in different stages of development.^10^ Long-term follow-up data from larger cohort of EoE patients will be revealing and can help provide further guidance for use of dupilumab and other biologics to be used in clinical practice.
